# First Description of Colistin and Tigecycline-Resistant *Acinetobacter baumannii* Producing KPC-3 Carbapenemase in Portugal

**DOI:** 10.3390/antibiotics7040096

**Published:** 2018-11-06

**Authors:** Cátia Caneiras, Filipa Calisto, Gabriela Jorge da Silva, Luis Lito, José Melo-Cristino, Aida Duarte

**Affiliations:** 1 Microbiology and Immunology Department, Interdisciplinary Research Centre Egas Moniz (CiiEM), Faculty of Pharmacy, Universidade de Lisboa, 1649-003 Lisboa, Portugal; fcalisto@itqb.unl.pt (F.C.); aduarte@ff.ulisboa.pt (A.D.); 2 Institute of Environmental Health (ISAMB), Faculty of Medicine, Universidade de Lisboa, 1649-028 Lisboa, Portugal; 3 Instituto de Tecnologia Química e Biológica António Xavier, Universidade Nova de Lisboa, 2780-157 Oeiras, Portugal; 4 Faculty of Pharmacy, Universidade de Coimbra, Polo das Ciências da Saúde, Azinhaga de Santa Comba, 3000-548 Coimbra, Portugal; gjsilva@ci.uc.pt; 5 Laboratory of Microbiology, Centro Hospitalar Lisboa Norte, 1649-035 Lisboa, Portugal; lmlito@chln.min-saude.pt (L.L.); melo_cristino@medicina.ulisboa.pt (J.M.-C.); 6 Institute of Microbiology, Institute of Molecular Medicine, Faculty of Medicine, Universidade de Lisboa, 1649-028 Lisboa, Portugal

**Keywords:** antimicrobial resistance, Gram-negative bacteria, *K. pneumoniae*, *A. baumannii*, KPC-3 carbapenemase, colistin, tigecycline

## Abstract

Herein, we describe a case report of carbapenem-resistant *Acinetobacter baumannii* and *Klebsiella pneumoniae* isolates that were identified from the same patient at a Tertiary University Hospital Centre in Portugal. Antimicrobial susceptibility and the molecular characterization of resistance and virulence determinants were performed. PCR screening identified the presence of the resistance genes *bla*_KPC-3_, *bla*_TEM-1_ and *bla*_SHV-1_ in both isolates. The KPC-3 *K. pneumoniae* isolate belonged to the ST-14 high risk clone and accumulated an uncommon resistance and virulence profile additional to a horizontal dissemination capacity. In conclusion, the molecular screening led to the first identification of the *A. baumannii* KPC-3 producer in Portugal with a full antimicrobial resistance profile including tigecycline and colistin.

## 1. Introduction

The acquisition and emergence of carbapenem resistance among Gram-negative bacteria (GNB) is a major cause of concern since carbapenems currently represent the treatment of choice for severe infections caused by multidrug-resistant (MDR) strains producing extended-spectrum β-lactamases (ESBL) which is a major global challenge in the treatment of these pathogens [1]. The carbapenemases frequently detected in *Enterobacteriaceae* are: (i) class A β-lactamases (e.g., *K. pneumoniae* carbapenemase; KPC); (ii) class B β-lactamases/metallo-β-lactamases (e.g., New Delhi metallo-β-lactamase-1; NDM-1) and (iii) class D β-lactamases (e.g., oxacillinase-48; OXA-48-like carbapenemases) [2,3]. Several reports have identified these plasmid-encoded carbapenemases worldwide but their prevalence varies geographically [2]. In 2017, the World Health Organization published a global priority pathogen list of antibiotic-resistant bacteria to help in prioritizing the research and development of new and effective antibiotic treatments. In this list, carbapenem-resistant *Enterobacteriaceae* and *Acinetobacter baumannii* are identified as two of the top three critical threats [4]. Antimicrobial resistance and bacterial virulence have developed on different timescales but they share some common characteristics and studies regarding the interplay between these factors are needed. Additionally, the development of new strategies involving new antimicrobial compounds, novel diagnostic methods that focus on high-risk clones and rapid tests to detect virulence markers may help to resolve the increasing problem of the association between virulence and resistance, which is becoming more beneficial for pathogenic bacteria with consequent therapeutic inefficacy [5]. Although great efforts have been made to enhance epidemiological surveillance in Europe, the detection of virulence traits and the molecular characterization of carbapenem-resistant isolates from some countries remain scarce.

This article aims to describe a case report of carbapenem-resistant *A. baumannii* and *K. pneumoniae* isolates that were identified from the same patient at a Tertiary University Hospital Centre in Portugal, leading, to the best of our knowledge, to the first description of the *A. baumannii* KPC-3 producer in Portugal.

## 2. Results

A 35-year-old Portuguese Caucasian female patient with a medical history of renal insufficiency was admitted to a Tertiary University Hospital Centre in Lisboa, Portugal at the beginning of January. At the end of the same month, the patient underwent gastro-enterotomy surgery. A carbapenem-resistant *K. pneumoniae* bacterial pathogen was identified in an infected wound at the beginning of February. One month later, a new surgery was done at the same general surgical ward. At the end of March, carbapenem-resistant *A. baumannii* was isolated from the same patient, also from an infected wound and in the same surgical department. Previous failed treatments with meropenem, linezolid and ciprofloxacin were documented. Considering the clinical instability of the patient, a prolonged hospitalization (from January to May) in the general surgery ward occurred. Despite all the efforts, the clinical condition worsened, an immunosuppression clinical state occurred and the patient died. Both clinical pathogens were preserved and sent to the Laboratory of Microbiology and Immunology in the Faculty of Pharmacy for specific and additional microbiological studies.

Carbapenem-resistant *K. pneumoniae* and *A. baumannii* were both recovered from the wound sample. After identification, the antimicrobial susceptibility profiling analysis indicated that the *K. pneumoniae* strain was resistant to all antibiotics tested, except tigecycline and colistin, while *A. baumanni* showed resistance to all antibiotics studied (Table 1). Screening for carbapenemase yielded positive results when using the Modified Hodge test.

PCR screening for β-lactamase genes followed by DNA sequencing identified the presence of the resistance genes *bla*_KPC-3_, *bla*_TEM-1_ and *bla*_SHV-1_ in both isolates. The *OmpK35* and *OmpK36* porin genes were positive in the *K. pneumoniae* strain and no mutational changes were found by DNA sequencing. Multilocus sequence typing (MLST), based on the analysis of internal fragments of seven housekeeping genes (*gapA*, *infB*, *mdh*, *pgi*, *phoE*, *rpoB* and *tonB*) revealed that the *K. pneumoniae* clinical isolate belonged to sequence type 14 (ST-14). Additionally, *K. pneumoniae* virulence factors were assessed by PCR with specific primers for the K2 serotype, fimbrial adhesins type 1 and type 3, haemolysin, aerobactin, mucoid regulator and the hypermucoviscosity phenotype. All except the mucoid and hypermucoviscosity phenotype virulence factors were identified (Table 2).

In order to study the transferability of the resistance profile, biparental mating between the *K. pneumoniae* isolate and the *E. coli* strain J53AziR was conducted and a transconjugant strain was selected. Replicon typing classified this plasmid within the incompatibility group IncFrepB. The transconjungant strain showed a susceptibility profile similar to the donor strain, with resistance to amoxicillin/clavulanic acid, cefotaxime and ceftazidime, whereas the carbapenems and cefoxitin showed decreased susceptibility, since they were not under the influence of porins. The genetic environment of the *bla*_KPC-3_ gene was characterized, namely, we searched for the genes associated with Tn*4401*, a Tn3-based transposon involved in *bla*_KPC_ gene mobilization transposon, in the *K. pneumoniae* and *A. baumannii* isolates. The Tn*4401b* transposon was identified in both isolates. The plasmid incompatibility group IncFrepB was also identified in the *A. baumannii* isolate.

## 3. Discussion

*K. pneumoniae* is the causative agent of several different healthcare-associated infections, such as wound infections, bloodstream infections, meningitis and pneumonia [6]. The extensive use of antimicrobials has led to a high incidence of resistance [7]. In our study, the firstly identified *K. pneumoniae* isolate showed a multidrug resistance profile to all β-lactams (including carbapenems) but also to aminoglycosides and fluoroquinolones. Tigecycline, colistin and carbapenem were the most commonly used drugs in combination antibiotic treatment in carbapenem-resistant infections [7]. However, carbapenemase-producing *A. baumannii*, which was identified three months later, was resistant to all antibiotics studied, limiting treatment options. In Portugal, the carbapenem resistance rates in *K. pneumoniae* increased from 0.9% (2009) to 5.2% (2016) and a worryingly resistance rate of 51.9% (2016) was reported for *A. baumannii* isolates, [8] despite the reduction trends in carbapenem antimicrobial consumption (ESAC-Net) [9].

Regardless of the efforts, our patient died. Pang et al. studied the prevalence and treatment for carbapenem-resistant *Enterobacteriaceae* infections in three tertiary care hospitals and showed poor mortality outcomes (23% at 28 days) but an effective treatment with the quinolone antibiotic [10]. Worryingly, infection with carbapenem-resistant *A. baumannii* is associated with a risk of mortality that is twice that of infection with its carbapenem-susceptible counterparts [11] as the high patient mortality rate (44% at 28 days) found in the AIDA trial demonstrated [12]. So, it is critical to effectively treat the primary infection in order to avoid co-infections or secondary infections with more resistant pathogens with consequent therapeutic failure. The role of old antibiotics in the era of antibiotic resistance should be promoted, such as the case of fosfomycin, which may be indicated for infections of the central nervous system, soft tissues, bone, lungs and abscesses due to its extensive tissue penetration [13,14].

The outer membrane of Gram-negative bacteria is a unique architecture that acts as a potent permeability barrier against toxic molecules, such as antibiotics [15]. It has been reported that a loss of porins OmpK35 and OmpK36 led to an increase in carbapenem and ciprofloxacin resistance [16]. DNA sequence analyses and protein homology searches were conducted and no changes were found when compared with *K. pneumoniae* isolates (GenBank accession number AJ303057 and GU461279), which is in accordance with studies described by other authors, namely in wound specimens [6] and KPC-3 producers [17], with the results being indicative that carbapenemase production is the main carbapenem resistance mechanism.

The genetic characterization confirmed the phenotypic features described since it identified the gene coding of the carbapenemase KPC-3 in co-expression with broad-spectrum β-lactamases (TEM-1 and SHV-1). Also, the *A. baumannii* showed the same resistance profile. The most common carbapenemase described worldwide is KPC-2 [18,19,20,21,22] but KPC-3 has already been identified in the United States [23], Israel [22], Italy [24] and Spain [25]. In Portugal, the first carbapenem-resistant *K. pneumoniae* was identified in 2009 [26] and since then, dissemination to other *Enterobacteriaceae* [27] and the increasing frequency of hospital outbreaks [28] has led to the creation of the Epidemiological Surveillance of Antimicrobial Resistance Guidelines, which contains mandatory notification of these pathogens [29].

The genetic environment of the *K. pneumoniae* strain was determined in order to understand if there had been a horizontal spread of the *KPC-3* gene between the *K. pneumoniae* and *A. baumannii* isolates. Our study describes a horizontal dissemination ability of the *bla*_KPC-3_ gene found in the *K. pneumoniae* isolate by an identical mobile genetic element, the Tn*4401b* isoform which is associated with a high resistance to carbapenems [17,30], propagated by a single type of plasmid, IncFrepB. The *K. pneumoniae* and *A. baummanii* isolates found in the same patient shared the same IncFrepB replicon origin which is indicative of a potential horizontal dissemination between these distinct species [31,32]. Additional studies should clearly demonstrate the interspecies transfer of *bla*_KPC-3_ by whole-plasmid sequencing.

Type 1 fimbriae is the most common adhesin in *Enterobacteriaceae* and can lead to persistent urinary tract infections [33]. Type 3 fimbrial adhesins mediate the binding of *K. pneumoniae* to endothelial and epithelial cells of the urinary and respiratory tracts. Many *K. pneumoniae* clinical isolates express both type 1 and type 3 fimbrial adhesins [33,34] but interestingly, in the current study, we found the coding genes to both of these adhesive structures but also to the K2 capsular serotype, which is predominantly associated with virulent strains [35], the iron siderophore aerobactin and hemolysin virulence factors.

Wasfi et al. demonstrated that only 7% of MDR *K. pneumoniae* isolates have the K2 capsular genotype [6]. The hemolysin virulence factor was detected in enterohemorrhagic *Escherichia coli* [36] and recently, also in uropathogenic bacteria, where has been described as causing programmed cell necrosis by altering mitochondrial dynamics [37]. The aerobactin mediates the acquisition of iron to help virulent bacteria to overcome iron starvation while bacteria invade and proliferate in the human system [38]. Russo et al. showed that aerobactin accounts for increased siderophore production, resulting in a 100% mortality rate and demonstrating the virulence of the isolates and their ability to cause infection at a low dose [39]. The virulence gene *aerobactin* has been previously detected in *Enterobacteriaceae* [40] including carbapenem-resistant *K. pneumoniae* isolates [6].

The carbapenemase producers are usually associated with highly resistant but low virulent strains [40,41,42]. In Brazil, De Cassia Andrade et al. reported the accumulation of virulence genes of KPC-2 *K. pneumoniae* isolates, along with the multi-resistance profile [43]. Also, in the United States, Krapp et al. described one *K. pneumoniae* KPC-3, SHV-28 and OXA-9 producer with the following virulence genes: enterobactin (*entABCDEF*), aerobactin receptor (*iutA*), type 1 and 3 fimbrial adhesion genes and the salmochelin receptor (*iroN*) [44]. These studies align with our findings in Portugal and suggest that the carbapenem-resistant *K. pneumoniae* strains are increasing in virulence. However, considering that we only described one clinical situation with one *K. pneumoniae* isolate, additional studies should be promoted, specifically regarding the interplay between resistance and virulence in *K. pneumoniae*. However, of note, our preliminary results indicate that variability in virulence profiles can exist according to the geographic origin of the isolate.

The MLST International clone ST-258 has been recognized as the prevalent ST of carbapenem-resistant *K. pneumoniae* isolates worldwide [45,46,47,48]. Herein, we described an isolate that belongs to sequence type ST-14 which has been associated with pan resistant isolates and the production of OXA-48 and NDM carbapenemases with a higher colistin rate of resistance when compared with isolates outside the cluster (37.1% vs. 27.1%) [49]. We should continuously highlight the importance of monitoring the emergence of highly virulent and resistant *K. pneumoniae*.

Future research regarding the colistin resistance molecular mechanisms in Gram-negative bacteria is needed. Furthermore, additional studies exploring the dangerous connections between resistance and virulence in Gram-negative infections and their impact on therapeutic efficacy should be incentivized.

## 4. Materials and Methods

### 4.1. Bacterial Isolates

The isolates were recovered using standard clinical operating procedures. Bacterial identification and antimicrobial susceptibility testing were performed at the microbiology laboratory by automated systems (Vitek2^®^, BioMérieux, Marcy, l’Etoile, France) and confirmed by the disk diffusion test in accordance with the methodology of the European Committee on Antimicrobial Susceptibility Testing (EUCAST), available at the European Society of Clinical Microbiology and Infectious Diseases (ESCMID) website (http://www.eucast.org/ast_of_bacteria/disk_diffusion_methodology/). Isolates with reduced susceptibility to carbapenems were selected and send to the Microbiology and Immunology Department in the Faculty of Pharmacy for specific and additional microbiological studies. The isolates were held in stock frozen in brain heart infusion (BHI) broth (VWR Prolabo^®^, Lisboa, Portugal) with 15% glycerol at −80 °C. For the analysis, the strains were grown in BHI broth for 18 h at 37 °C and seeded in Lysogeny broth (LB), more commonly known as Luria–Bertani agar (VWR Prolabo^®^, Lisboa, Portugal). Both isolates were recovered from wound infections.

### 4.2. Antimicrobial Susceptibility Testing

Bacterial antimicrobial susceptibility testing was performed in accordance with the EUCAST standardized disk diffusion method in Mueller–Hinton (MH) agar medium (VWR Prolabo^®^, Lisboa, Portugal). The detailed methodology and the preparation and storage of MH agar are described in the EUCAST Disk Diffusion Method for Antimicrobial Susceptibility Testing guidelines, which are available at (http://www.eucast.org/ast_of_bacteria/disk_diffusion_methodology/)*.* Quality control was carried out in accordance with EUCAST (version 6.0, 2016) and the Clinical and Laboratory Standards Institute (CLSI) guidelines (M100-S20), namely, *Escherichia coli* ATCC 25922 and *Escherichia coli* ATCC 35218 were used as controls for the inhibitor component of beta-lactam inhibitor-combination disks. Susceptibility was tested by a panel of antibiotics: amoxicillin/clavulanic acid (20/10 µg), cefoxitin (30 µg), cefotaxime (5 µg), ceftazidime (10 µg), imipenem (10 µg), gentamicin (10 µg), ciprofloxacin (5 µg) and tigecycline (15 µg). The inhibition zones were interpreted in accordance with EUCAST. The isolates were categorized as susceptible, standard dosing regimen (S); susceptible, increased exposure (I); and resistant (R) by applying the breakpoints in the phenotypic test results. Multidrug-resistant (MDR) bacteria were defined as those that acquired non-susceptibility to at least one agent in three or more antimicrobial categories, in accordance with the United States Center for Disease Control and Prevention (CDC) and the European Centre for Disease Prevention and Control (ECDC) consensual definition [50].

### 4.3. Resistance and Virulence Determinants

PCR-based screening for the most commonly found β-lactamase families was performed with specific primers that have already been described (*bla*_SHV_ [51], *bla*_DHA_ [52], *bla*_CMY_ [53], *bla*_CTX-M_ [54]) including carbapenemase genes (*bla*_KPC_ [55], *bla*_IMP_ [56], *bla*_VIM_ [57] and *bla*_OXA_ [58]). The virulence factors were assessed by PCR with specific primers for the K2 serotype (*K2A*), fimbrial adhesins type 1 (*fimH*) and type 3 (*mrkD_v1_* and *mrkD_v2–4_*), haemolysin (*khe*), aerobactin (*iucC*), regulator of mucoid phenotype (*rmpA*) and the hypermucoviscosity phenotype (*magA*). The primers for *bla*_TEM_, *bla*_NDM_, *OmpK35* and *OmpK36* and for virulence genes were designed in our laboratory in accordance with the sequences’(5′–3′) available on Genbank (Table 3).

### 4.4. Molecular Methods

Polymerase chain reactions (PCRs) were performed using the commercial kit puReTaq Ready-To-Go PCR Beads (GE Healthcare^®^, Lisboa, Portugal) in accordance with the manufacturer’s instructions. Subsequently, the PCR products were resolved in 1% agarose gel in 1× concentrated Tris-Borate-EDTA (TBE) buffer (Sigma-Aldrich^®^, Lisboa, Portugal) (89 mM Tris-borate and 2 mM EDTA) and stained with GelRed (Biotium^®^, Lisboa, Portugal). Positive and negative controls were included in all PCR assays. The positive controls used were positive strains from the Laboratory of Microbiology collection that had been sequenced previously and the negative controls were provided by the PCR commercial kit puReTaq Ready-To-Go PCR Beads. The resulting PCR products were submitted to purification using the JETquick Spin Column Technique PCR Purification Kit (Genomed^®^, Lisboa, Portugal), in accordance with the producer’s instructions and were sequenced at Macrogen Korea and STABVida Portugal. Searches for nucleotide sequences were performed with the BLAST program, which is available at the National Center for Biotechnology Information website (http://www.ncbi.nim.nih.gov/). Multiple-sequence alignments were performed with the ClustalX program, which is available at the European Bioinformatics Institute website (http://www.ebi.ac.uk/Tools/msa/clustalw2).

### 4.5. Transfer of blaKPC-3 and Plasmid Characterization

The identification of the incompatibility groups of plasmids was performed by the Replicon Typing technique [59]. This technique allowed us to identify the origins of replication of plasmids belonging to different incompatibility groups (*IncHI1*, *IncHI2*, *IncI1-I*, *IncX*, *IncL/M*, *IncN*, *IncFIA*, *IncFIB*, *IncW*, *IncY*, *IncP*, *IncFIC*, *IncA/C*, *IncT*, *IncFIIAs*, *IncK*, *IncB/O*, *IncF*). Subsequently, the transfer of the *bla*_KPC-3_ gene to the *E. coli* J53 resistant azide (AziR) receptor was performed by conjugation [60]. The transconjugants were selected in Müller–Hinton agar (VWR Prolabo^®^) supplemented with sodium azide (100 μg/mL) and cefotaxime (1 μg/mL).

### 4.6. Multilocus Sequence Typing (MLST)

MLST was performed on the *K. pneumoniae* isolate as previously described [61]. The sequence was performed at Macrogen Korea and submitted to the MLST database for allele attribution. The *K. pneumoniae* database is available at the Pasteur MLST website (http://www.pasteur.fr/mlst/) and was last accessed on 2 May 2018.

### 4.7. Ethical Approval

Isolates were obtained as part of routine diagnostic testing and were analysed anonymously. All data were collected in accordance with the European Parliament and Council Decision on the Epidemiological Surveillance and Control of Communicable Disease in the European Community. Clinical and epidemiological data were collected from clinical records. The study proposal was also approved by the Research Ethics Committee of the Faculty of Medicine, University of Lisboa, Portugal.

## 5. Conclusions

In conclusion, we identified the first KPC-3 carbapenemase-producing *A. baumannii* isolate in Portugal associated with a fateful opportunistic infection preceded by a highly resistant and virulent *K. pneumoniae* KPC-3 producer belonging to the ST-14 high-risk clone. This illustrates a previously undescribed situation in our country with significant impact regarding the therapeutic antibiotics available for severe infections. The knowledge of specific genotyping patterns, resistance and virulence determinants of pathogens is crucial for the development of new antibacterial agents and adjuvants against antimicrobial resistant Gram-negative bacteria.

## Figures and Tables

**Table 1:** Phenotypic characterization of the *K. pneumoniae* an-d *A. baumannii* isolates.

Classes of Antibiotics	List of Antibiotics ^1^ (*n* = 15 Agents)	*K. pneumoniae* 69633	*A. baumannii*^4^ 86982
Penicillins	Ampicillin	R	R
β-lactam/β-lactamase inhibitor combinations	Amoxicillin-clavulanic acid	R	R
	Piperacillin-tazobactam	R	R
Cephalosporins	Cefoxitin-C2G ^2^	R	R
	Cefotaxime-C3G ^3^	R	R
	Ceftazidime-C3G ^3^	R	R
Monobactams	Aztreonam	R	R
Carbapenems	Imipenem	R	R
	Meropenem	R	R
	Ertapenem	R	R
Aminoglycosides	Gentamicin	R	R
Fluoroquinolones	Ciprofloxacin	R	R
	Levofloxacin	R	R
Polymyxins	Colistin	S	R
Tetracyclines	Tigecycline	S	R

^1^ β-lactam antibiotics classes are shaded grey. Red/R indicates resistance and green/S indicates susceptible, standard dosing regimen. Strains were recovered from the same patient. ^2^ C2G: second generation cephalosporin; ^3^ C3G: third generation cephalosporin ^4^ *A. baumannii* is considered to be intrinsically resistant to ampicillin, cefotaxime, aztreonam and ertapenem.

**Table 2:** Resistance and virulence molecular characteristics of *K. pneumoniae* carbapenemase (KPC)-3 producer isolates.

Strain	β-Lactamases Identified	PBRT ^1^	MLST	Virulence Profile
*K. pneumoniae* 69633	KPC-3 + SHV-1 + TEM-1	IncFrepB	ST-14	K2 + fimH + mrkDV1 + mrkDV2-4 + khe + iucC
*A. baumannii* 86982	KPC-3 + SHV-1 + TEM-1	IncFrepB	-	-

^1^ Legend: PBRT: PCR-based replicon typing; MLST: multilocus sequence typing.

**Table 3:** List of primer designs in the current study and expected amplicon size.

Gene	DNA Sequence (5′ to 3′)	Amplicon Size (bp)	EMBL Accession Number (Genbank)
*bla*_NDM_	F: TATCGCCGTCTAGTTCTGCTG	871	AB604954
	R: ACTGCCCGTTGACGCCCAAT		
*K2A*	F: CAACCATGGTGGTCGATTAG	531	EF221827
	R: TGGTAGCCATATCCCTTTGG		
*fimH*	F: TGTTCACCACCCTGCTGCTG	512	NC_012731.1
	R: CACCACGTCGTTCTTGGCGT		
*mrkD_V1_*	F: CGGTGATGCTGGACATGGT	300	EU682505.2
	R: CCTCTAGCGAATAGTTGGTG		
*mrkD_V2–4_*	F: CTTAATGGCGMTGGGCACCA	950	AY225463.1
	R: TCATATGCGACTCCACCTCG		AY225464.1
			AY225465.1
*khe*	F: TGATTGCATTCGCCACTGG	428	NC_012731.1
	R: GGTCAACCCAACGATCCTGG		
*iucC*	F: GTGCTGTCGATGAGCGATGC	944	NC_005249.1
	R: GTGAGCCAGGTTTCAGCGTC		
*rmpA*	F: ACTGGGCTACCTCTGCTTCA	516	NC_012731.1
	R: CTTGCATGAGCCATCTTTCA		
*magA*	F: TCTGTCATGGCTTAGACCGAT	1137	NC_012731.1
	R: GCAATCGAAGTGAAGAGTGC		
*ompK35*	F: ATATTCTGGCAGTGGTGATCC	1012	AJ303057
	R:GCTTTGGTGTAATCGTTGTC		
*ompK36*	F: TAGCAGGCGCAGCAAATGC	1031	GU461279
	R: TGCAACCACGTCGTCGGTA		

Legend: F—forward primer; R—reverse primer.
